# Georeferenced LiDAR 3D Vine Plantation Map Generation

**DOI:** 10.3390/s110606237

**Published:** 2011-06-09

**Authors:** Jordi Llorens, Emilio Gil, Jordi Llop, Meritxell Queraltó

**Affiliations:** Department of Agri Food Engineering and Biotechnology, Universitat Politècnica de Catalunya Campus del Baix Llobregat, Edifici D4, Esteve Terradas, 8 08860- Castelldefels, Spain; E-Mails: Jordi.Llorens.Calveras@upc.edu (J.L.); Jordi.Llop-casamada@upc.edu (J.L.); Meritxell.Queralto@upc.edu (M.Q.)

**Keywords:** LiDAR, canopy density, vineyard, GPS, UTM coordinates

## Abstract

The use of electronic devices for canopy characterization has recently been widely discussed. Among such devices, LiDAR sensors appear to be the most accurate and precise. Information obtained with LiDAR sensors during reading while driving a tractor along a crop row can be managed and transformed into canopy density maps by evaluating the frequency of LiDAR returns. This paper describes a proposed methodology to obtain a georeferenced canopy map by combining the information obtained with LiDAR with that generated using a GPS receiver installed on top of a tractor. Data regarding the velocity of LiDAR measurements and UTM coordinates of each measured point on the canopy were obtained by applying the proposed transformation process. The process allows overlap of the canopy density map generated with the image of the intended measured area using Google Earth^®^, providing accurate information about the canopy distribution and/or location of damage along the rows. This methodology was applied and tested on different vine varieties and crop stages in two important vine production areas in Spain. The results indicate that the georeferenced information obtained with LiDAR sensors appears to be an interesting tool with the potential to improve crop management processes.

## 1. Introduction

Electronic measurements of canopy characteristics appear to be the most accurate method of providing reliable and objective information regarding the intended target during pesticide application. The variable rate application concept [1–3] requires that the sprayer be updated with electronic equipment able to measure, save and manage a large amount of information relative to the canopy geometry. This information will then be used either in real time to instantaneously modify the working parameters of the sprayer [2,4,5] or in a post process way to develop canopy maps to be used as a decision making tool in further agronomic applications [6–9]. In the last few years, different electronic canopy measurement procedures have been developed. Some researchers have proposed comparative measurements of canopy structure using ultrasonic or LiDAR (Light Detection and Ranging) sensors [10–13]. In all cases, LiDAR appears to be the most accurate procedure for canopy measurements. Specific problems in the use of ultrasonic sensors have been noted [14–16], mainly due to the influence of weather conditions or the interference produced by multiple sensor operations.

According Lee *et al.* [17] the retrieval of tree and forest structural attributes from LiDAR data has focused largely on utilising canopy height models, but these have proved only partially useful for mapping and attributing stems in complex, multi-layered forests. For that reason this research group developed a new index, termed the Height-Scaled Crown Openness Index (HSCOI), which provides a quantitative measure of the relative penetration of LiDAR pulses into the canopy. Lee *et al*. [17] concluded that the HSCOI was developed to maximize the amount of information that can be retrieved from scanning LiDAR and its use facilitated the location, density and height of tree stems associated with both the upper and sub-canopy strata. Goodwin *et al*. [18] used a LiDAR for the assessment of (eucalyptus tree) forest structures in Australia. In their conclusions the authors demonstrate that LiDAR data can be used to map the structural variation in eucalyptus forests with different proportions of tree development stages. Estimated crown volume as derived from ground measurements and LiDAR data were shown to be highly correlated with an R^2^ of 0.79 at the plot scale.

Van der Zande *et al.* [19] used a commercially available and inexpensive LiDAR (a SICK LMS 200) for canopy characterization on artificial trees and concluded that the sideway lateral measurement pattern was the most appropriate measuring method to describe the structural aspects of the artificial tree. Moorthy *et al*. [20] proposed a methodology for olive tree crown characterization using an Intelligent Laser Ranging and Imaging System (ILRIS-3D). From the observed 3D laser pulse returns, quantitative retrievals of tree crown structure and foliage assemblage were obtained. Robust methodologies were developed to characterize diagnostic architectural parameters, such as tree height, crown width, crown height, crown volume and Plant Area Index (PAI) which are not easily obtained parameters with traditional *in situ* methods.

Palacín *et al.* [21] proposed a method to estimate leaf surface and canopy volume after LiDAR measurements that used the diameter and position of each laser spot in the canopy. In their study, the relationship between canopy volume and leaf surface was generated using the total canopy measurements, not individually for each crop slice, defined as the vertical outline of the vine for the current position of the LiDAR. The mathematic relationship obtained is interesting and can be used in further LiDAR measurements in other circumstances.

Canopy characterization using LiDAR has been also proposed in studies of orchards and vine plantations [5,22,23]. In these studies, one of the greatest challenges was identifying the correct and precise procedure to join measurements from the left and right side of the crop. Different tedious and difficult methodologies have been proposed to enable the correct synchronization between left and right measurements. These methods include placing reference elements on exact points in the row, allowing one to obtain a spot cloud representing the canopy resulting in complicated data management. Further developments have improved this process. For example, an automated system has been developed [24]. In this automated system, the spot cloud was processed using specific software that automatically overlapped the data. This software was developed in VBA (Visual Basic for Applications) using AUTOCAD (Autodesk, Inc.).

The use of GPS has been implemented in other studies together with electronic devices for canopy characterization. For example, Zaman *et al.* [14] added a GPS receiver to their sprayer to control and monitor the tractor speed during canopy scanning using ultrasonic sensors in citrus plantations. Schumann *et al.* [25] used a Trimble AgGPS 132 with differential correction in an attempt to georeference the measurements obtained from ten ultrasonic sensors in citrus plantations. Synchronized information obtained using ultrasonic sensors and DGPS allowed georeferenced maps of canopy volume or tree height maps to be obtained. Further development of this research [26] allowed the elaboration of maps containing tree dimensions measured using LiDAR sensors. The generated maps were subsequently used to define a differential fertilizer program.

Generation of canopy maps and their further use in the improvement of different agronomy procedures in fruit and vine plantations has been the objective of several research groups, who have had varied success. Shimborsky [6] proposed a canopy characterization method that employed the configuration of canopy maps using satellite images together with detailed digital information regarding the land. The final product of their method provides a level line curves map on which the geometrical shape of the trees can be observed. However, this method is difficult, expensive and not directly applicable to current fruit or vine plantations.

This study was conducted to generate a georeferenced canopy map of measured vine plantations using measurements collected generated by a combined GPS and LiDAR system. The main objective of this study can be summarized in the following partial objectives:

- Configuration of a LiDAR and GPS system for canopy scanning.
- Development of a proper methodology to georeference the frequency of LiDAR returns and its relation with canopy characteristics
- Validation of the methodology in different vine plantations and situations.

## 2. Materials and Methods

### 2.1. LiDAR Sensor

A LMS-200 LiDAR (Sick, Düsseldorf, Germany) was used in this study. This LiDAR is a fully-automatic divergent laser scanner based on measurement of the time-of-flight (TOF) with an accuracy of ±15 mm in a single shot measurement and a 5 mm standard deviation in a range of up to 8 m [21]. The time between the transmission and reception of the pulsed near-infrared laser beam is used to measure the distance between the scanner and the reflecting object surface. The laser beam is deflected by a rotating mirror turning at 4,500 rpm (75 rps), which results in a fan shaped scan pattern in which the maximum scanning angle is 180°. The angular resolution can be set to l°, 0.5°, or 0.25°, making 181, 361 and 400 measurements, respectively, at full scanning range with a response time of 13, 26 and 53 ms, respectively. The LMS-200 has a standard RS232 serial port for data transfer, which can be set to 9.6, 19.2 or 38.4 Kbaud. The selected configuration during the field tests conducted for this study was: angular resolution 1°range of 180°and data transfer 38.4 Kbaud. These characteristics enabled the best resolution to be obtained during the scanning process.

### 2.2. Definition of Grid Resolution for LiDAR Measurements

**Figure 1.**
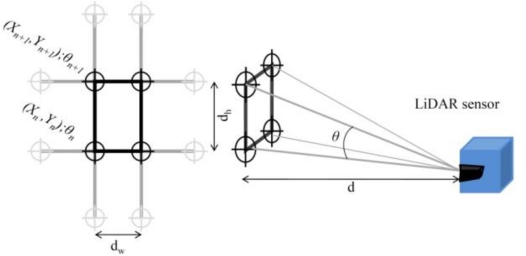
Definition of the reference grid for LiDAR measurements.

Before any scan for canopy characterization, the grid resolution of the sensor must be defined. This aspect will allow further comparisons among results obtained under different conditions and/or with different sensors. In this study, the grid resolution was established according to Figure 1.

The dimensions of a single cell of the grid depend on the forward speed, distance from the LiDAR sensor to the measured point on the canopy and the measuring frequency of the sensor, which are defined in Equations (1) and (2):

(1) $$d_{h}=\left|\frac{\text{tg}(\theta_{\text{n}}-90)-\text{tg}(\theta_{\text{n}+1}-90)}{\text{d}}\right|$$

(2) $$d_{w}=\frac{\text{V}}{\text{f}}$$

where *d_h_* is the vertical distance (height) between two consecutive laser returns (m); *θ* is the angle of each measurement (°); *d* is the distance from the LiDAR to the reference surface (m); *d_w_* is the horizontal distance (width) between two consecutive scans (m); *V* is the forward speed (m·s^−1^); and *f* is the frequency of scanning (Hz).

The reference grid established for any sensor will affect the final resolution [19,27] and the interpretation of the spots on the map obtained. For narrow reference grids, the spot cloud density will be increased and comparison with other measurements could be erroneous. Similar reference grids have been defined previously [13,28,29].

### 2.3. GPS Receiver

**Figure 2.**
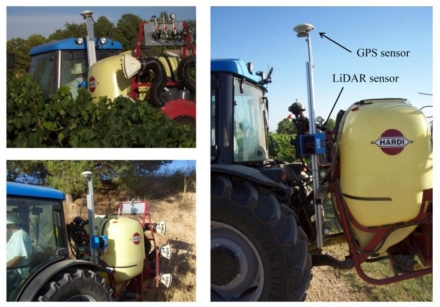
The LiDAR sensor and GPS receiver installed on the tractor for canopy measurements.

A Trimble AgGPS-132 DGPS antenna (Trimble Navigation Limited, Sunnyvale, CA, USA) and a LiDAR sensor were mounted on an intended stainless-steel mast placed between the tractor and the sprayer according to a previously described procedure [1,4,13] (Figure 2). GPS was configured to receive information at the maximum precision level using the differential correction from the EGNOS satellite (European Geostationary Navigation Overlay Service), which provides much more accurate position data and enhanced accuracy of speed determination than non corrected units [30]. Data regarding the forward speed and specific position on the field were acquired via serial port RS 232, using the National Marine Electronics Association (NMEA) 0183 protocol. The frequency of the data acquisition was 1 Hz.

### 2.4. GPS and LiDAR Communication

**Figure 3.**
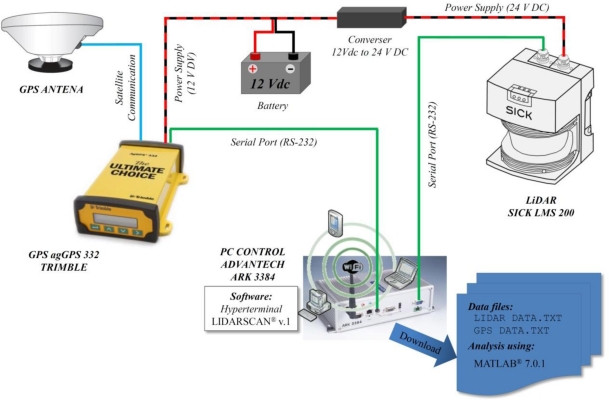
LiDAR, GPS receiver and computer. Scheme of communication.

The LiDAR sensor and GPS were both connected to a computer for data management and storage. Serial port RS 232 was used to connect both the GPS receiver and the LiDAR sensor to the computer. A power source (12 V DC for GPS and 24 V DC for LiDAR sensor) was used to supply energy to the system (Figure 3). The specific developed software, *LiDARScan v.1^®^*, based on VBA (Visual Basic for Applications) was used for LiDAR data management. In addition, the HyperTerminal tool (Windows^®^) was used to store the NMEA data sent by GPS. Synchronization of GPS and LiDAR was obtained using the starting time (in Universal Time Coordinated (UTC) format)) as a reference for the measurement process.

### 2.5. Field Measurements

**Figure 4.**
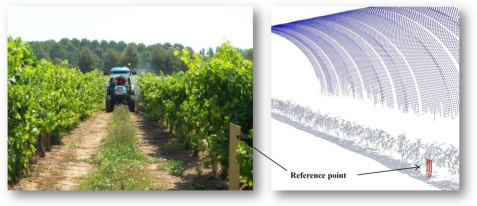
Measuring procedure: a LiDAR sensor placed on the mast reads the canopy on the left side of the tractor track.

The scanning process started when the tractor began the circulation between vine rows. On each track, vegetation on the left side of the tractor was scanned at 1.25 m·s^−1^ (4.5 km·h^−1^). The LiDAR position was checked and adjusted to place the sensor at 1.40–1.60 m over the ground so that the entire canopy could be scanned. Considering the measurement frequency of the LiDAR (10 Hz) assuming one single crop slice as individual measurement, and the tractor forward speed, the system was able to store and process 181 measurements every 0.1 s, which was equivalent to 0.125 m of displacement along the vine line. This scan process was repeated from the opposite side of the canopy to scan the other half of the vegetation. Figure 4 shows the measurement procedure with LiDAR.

Reference elements placed on exact points in the row can be observed as an example of the previous method used to assembly left and right measurements prior the use of the georeferencing process. The large curved object above the vines represents the laser beams that have not hit any object. Those lasers are represented, according the specifications of the sensor, as a curvy surface 8 m away from the LiDAR. Depending on the row line length, in some field tests it was necessary to restart the system at the end of the first track (left side) due to the large amount of data generated by the LiDAR sensor. During the whole process and due to the specific characteristics of the sensor, only single LiDAR returns were recorded for every measured point.

Field tests were conducted during Spring and Summer of 2009 and 2010 in fields containing different vine varieties—*Merlot* and *Cabernet Sauvignon*—during several crop stages (65, 75 and 85 according to the BBCH classification) [31]. Experiments were conducted in two different vine regions, Barcelona and Lleida (Spain).

### 2.6. Data Georeferencing Procedure

Once the two (*.txt) files were obtained (one from LiDAR and one from GPS), the georeferencing procedure started with data import from the GPS receiver (*.txt file) in NMEA format. This file was then transformed into UTM (Universal Transverse Mercator) coordinates using the $GPRMC (Recommended Minimum Specific GPS/TRANSIT Data) line. UTM is a coordinate system based on the cartographic projection that can be used to plot the entire mobile trajectory and the individual position of each data point obtained using LiDAR. Data transformation from NMEA to UTM was conducted according to the method proposed by Paggi *et al.* [32] using the WGS84 coordinate system.

**Figure 5.**
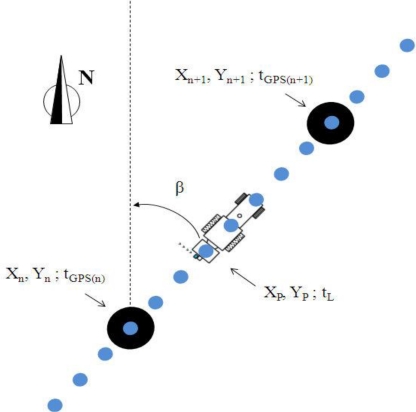
Procedure for determining the UTM coordinates of each LiDAR measurement point.

**Figure 6.**
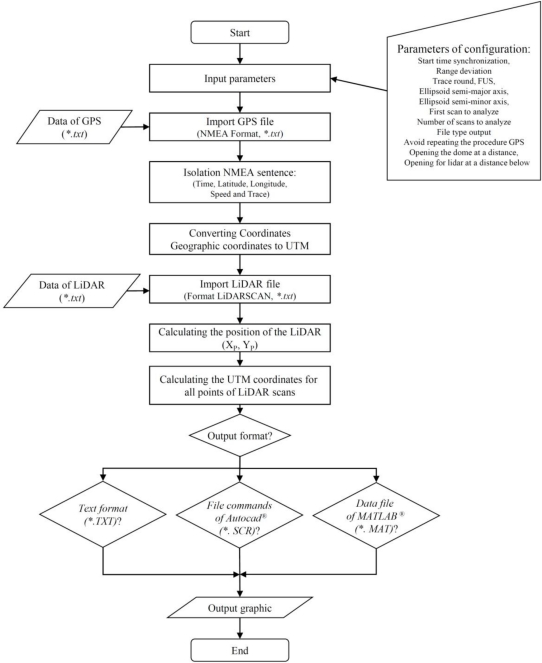
Flow chart of the first part of the georeferencing method.

The first step of the georeferencing procedure was to establish the position of the LiDAR sensor (Figure 5) on each scan slice (*X_P_*, *Y_P_*) and its relative position between two consecutive points with GPS measurements. The flow chart of this complex process is shown in Figure 6.

Points (*X_n_*, *Y_n_*) and (*X_n+1_*, *Y_n+1_*) (Figure 5) represent two consecutive points with UTM coordinates obtained by GPS. For each point, the measurement time (*t_GPS_*) was associated and registered. From this information, the values of *β*, which is the trajectory azimuth of the tractor on the row, and *d_p_*, which is the distance from the last UTM identified point to the (*X_p_*, *Y_p_*) position, were calculated according to Equations (3) and (4). The LiDAR measurement time (*t_L_*) was also calculated for each point:

(3) $$\beta =\text{arctg}\frac{\left({\text{X}}_{\text{n}+1}-\text{Xn}\right)}{\left({\text{Y}}_{\text{n}+1}-{\text{Y}}_{\text{n}}\right)}$$

(4) $$d_{p}=({\text{t}}_{\text{L}}-{\text{t}}_{\text{GPS}})\times \frac{\sqrt{{{(\text{X}}_{\text{n}}-{\text{X}}_{\text{n}+1})}^{2}+{\left({\text{Y}}_{\text{n}}-{\text{Y}}_{\text{n}+1}\right)}^{2}}}{{\text{t}}_{\text{GPS}(\text{n}+1)}-{\text{t}}_{\text{GPS}(\text{n})}}$$

**Figure 7.**
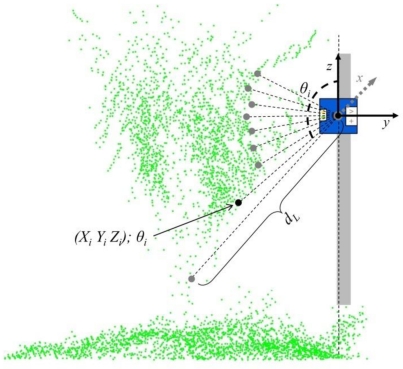
Spot cloud of measurements obtained using the LiDAR sensor. For each point, the georeferencing method allows the UTM coordinates to be defined. Green points represent the total measurement data for the canopy. Grey points represent values obtained in one scan.

After this process, the coordinates of each LiDAR measurement (*X_P_*,*Y_P_*) were calculated according to Equations (5) and (6). Those expressions allow determination of the exact position of the LiDAR sensor during each scan. Once the LiDAR position was calculated, the individual UTM coordinates of each measured point on the canopy were obtained (Figure 7). Values of (*Xi*, *Yi*, *Zi*; *θ_i_*) for each point measured in the canopy, *i* [0...180], were the obtained according to Equations (7), (8) and (9):

(5) $$X_{P}=X_{n}+(\text{cos}\beta +d_{P})$$

(6) $$Y_{P}=Y_{n}+(\text{sin}\beta +d_{P})$$

(7) $$X_{i}=X_{P}+\text{cos}\beta \times (d_{L}\times \text{sin}\theta_{i})$$

(8) $$Y_{i}=Y_{P}+\text{sin}\beta \times (d_{L}\times \text{sin}\theta_{i})$$

(9) $$Z_{i}=d_{L}\times \text{cos}\theta_{i}$$

### 2.7. Procedure of LiDAR returns Map Generation

**Figure 8.**
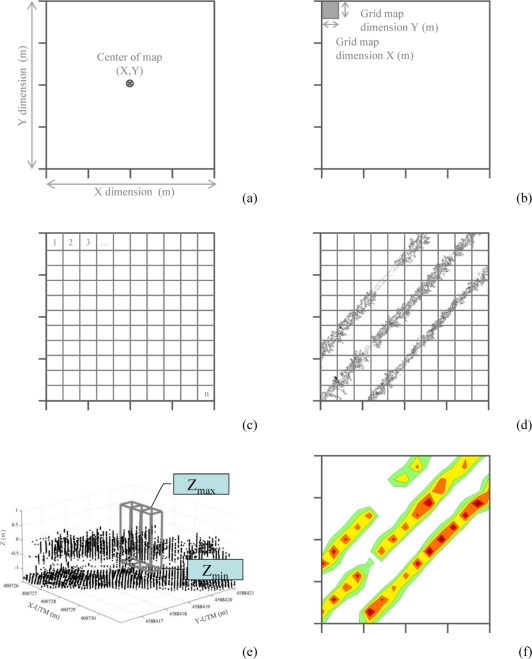
Graphical sequence of generation of the density map: **(a)** definition of mapping area characteristics; **(b)** grid definition; **(c)** mapped area divided according to the defined grid; **(d)** defined grid area with all measured points; **(e)** assignment of minimum and maximum height in each cell of the grid; **(f)** plot of map of density according to the established intervals.

After calculation of the coordinates of each point, the process of LiDAR returns density mapping was conducted to delimit the corresponding scanned area in the field. This area was defined by the UTM coordinates of its centre point and the two main dimensions (length and width; Figure 8(a)).

**Figure 9.**
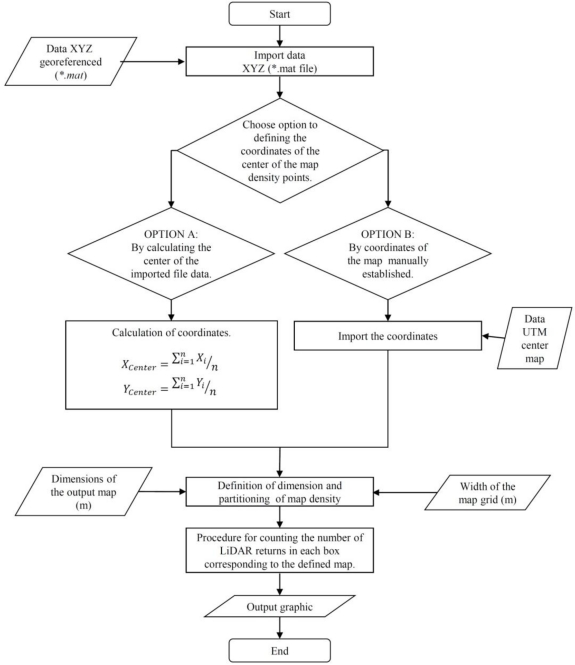
Flow chart of the second part of the georeferencing method.

The positioning of the centre point can be arranged using two different procedures: (a) automatically by calculating the average point of the UTM coordinates of all measured points obtained with LiDAR; or (b) by manual introduction of the centre point UTM coordinates. The process continues with the definition of the grid resolution that represents the real surface of a single data point. In this case, the map resolution was established in square units of 0.25 m^2^ (Figure 8(b,c)). Once the grid area was established, the system represented all of the measured points in a two dimensional layout (Figure 8(d)), and each square unit was linked to a defined range of measurements according to the maximum and minimum height (*Z_n_*) (Figure 8(e)). Finally, the map of the LiDAR returns densities was obtained and represented according to the selected levels of iso-density previously defined (Figure 8(f)). Figure 9 shows the flow chart of the entire canopy map generation process.

## 3. Results

Owing to a previous detailed georeferencing process, the system employed in the present study generates a text file with the UTM coordinates of each of the points measured on the canopy area with the LiDAR sensor. This file can be managed using some specific software able to represent 3D points (Autocad^®^, Matlab^®^) for the 3D representation of the canopy.

### 3.1. Georeferenced Density Map

**Figure 10.**
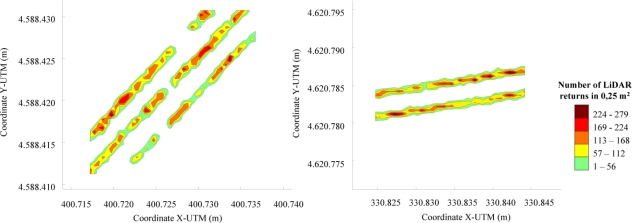
Georeferenced density map obtained after LiDAR measurement process on the field. Intervals have been defined according minimum and maximum LiDAR returns·cm^−2^ obtained in the field. Left: *Cabernet Sauvignon* (2008); right: *Merlot* (2009).

The georeferenced map could be associated with the canopy density map assuming a good correlation between laser returns and leaf area [13]. The georeferencing process developed in this research together with a five class density classification based on the number of LiDAR returns per surface unit allows representation of the measured row lines (Figure 10) with information regarding canopy presence and canopy distribution along the line. Gaps, zones with high leaf development, and zones with very low leaf area value can be precisely defined and placed into the exact position in the parcel.

Furthermore, once all of the spots were classified in their corresponding boxes, the procedure detailed on the flow chart shown in Figure 9 was applied to obtain a georeferenced grid map. A graphical interpretation of this step is show in Figure 10, in which a grid map of the spots cloud is transformed in a georeferenced map representing the rows of crops in the parcel.

**Figure 11.**
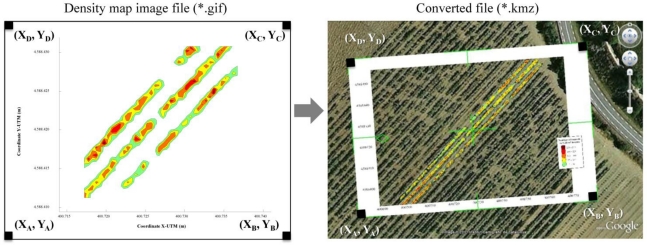
Procedure for the conversion of GIF files to KMZ files. This proposed method allows the density map over the image of measured field to be seen.

Once the density map was obtained, the UTM coordinates of each pixel of the four corners (A, B, C and D from Figure 11) of the (*GIF) file were defined. Applying a conversion process to this (*GIF) led to production of a new (*KMZ) file that was saved as a Google Earth^®^ compatible file. The use of Virtual Globes such as Google Earth and NASA Word Wind, and the production of high-quality Keyhole Markup Language (KML or its equivalent zipped KMZ) representations of scientific data has been widely described by [33]. Launching the (*.KMZ) file on a computer in which Google Earth^®^ had previously been installed enables the proposed application of the obtained maps allows to draw the generated density map exactly over the ortophotography of the measured parcel (Figure 11). Identification of gaps, leaf accumulation zones or other aspects related to of affecting canopy development can then be used in the crop management process.

**Figure 12.**
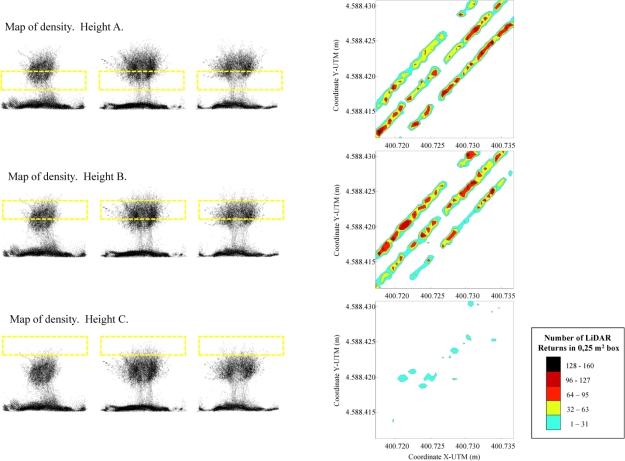
LiDAR return density obtained after LiDAR measurements can be split into the different heights obtained separately.

Other proposed applications of this density map are shown in Figure 12. This figure shows the partial canopy density for each level defined in the entire canopy. Spot clouds can be classified into each individual grid box according to their *Z_n_* coordinates, after which specific information about the canopy distribution based on height can be obtained and plotted. This information appears to be useful in processes based on the variable application rate in pesticide application in vineyards [1,4], allowing selection of different working parameters (*i.e.*, nozzle flow rate, air flow rate, air direction…) according to the non uniform canopy distribution.

### 3.2. Estimation and Georeferencing of Specific Canopy parameters

The interest of this new proposed method can be measured by its capability to estimate some canopy parameters and their geographical distribution along the field. A specific correlation between LiDAR return density and Leaf Area Index (LAI) was determined by [13], according Equation (10):

(10) $$LAI_{M}=0.0021\times I_{L}+0.137$$

where *LAI_M_* is the value of leaf area index manually measured (m^2^·m^−2^); and *I_L_* the amount of LiDAR return (returns·m^−1^).

**Figure 13.**
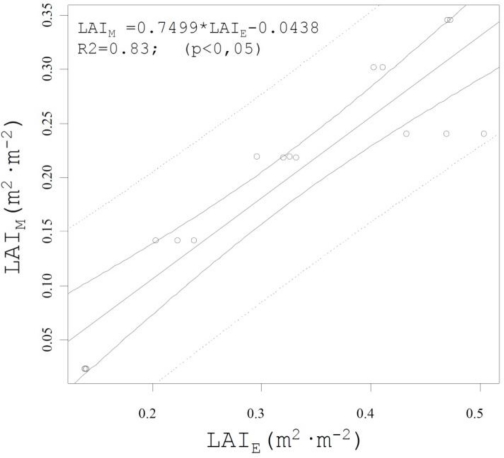
Relationship between estimated leaf area index (*LAI_E_*) obtained from LiDAR return density and leaf area manually measured (*LAI_M_*). Values represent all the varieties (*Cabernet Sauvignon* and *Merlot*) included in this research.

This correlation (R^2^ = 0.409) was determined with values obtained only with one single LiDAR pass (one side of the crop). Then, following the same procedure all the data (LiDAR returns) obtained in this research was managed and classified in four different canopy heights, dividing the whole canopy in individual zones of 0.40 m height. For every single cell of 0.40 m × 1.0 m length on the row the value of 50% of impact LiDAR returns obtained was calculated and transformed into a value representing the leaf area estimated (*LAI_E_*) applying Equation (10). Values of *LAI_E_* were calculated for all varieties and crop stages previously described and compared with manually measurements of LAI obtained for every single crop height [13]. Figure 13 represents the obtained correlation between estimated values (*LAI_E_*) and measured values (*LAI_M_*).

**Figure 14.**
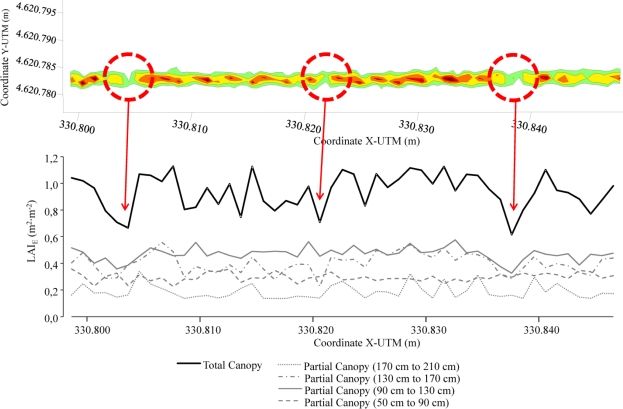
Evolution of LAI estimated values along the row crop obtained after density LiDAR returns classified on a 1.0 m length grid. Values have been obtained for the whole vegetation and for every single crop height previously defined (row 70 var*. Merlot*).

Once demonstrated the good correlation (R^2^ = 0.83) between the impact LiDAR return and values of measured LAI, the whole data package obtained with LiDAR can be managed and transformed into LAI values. In this sense, and just as an example of possibilities of the system, the total values of LiDAR returns corresponding to one specific crop line (row 70 var. *Merlot*) were arranged and classified into four different heights into the canopy (from 0 to 1.60 m every 0.40 m). Single boxes of 0.40 m × 1.0 m along the row crop were established and for every single one, the impact LiDAR return was converted into leaf area value applying the correlation obtained in Figure 13. This procedure leads to obtain the leaf area index variation along the row crop (general and individually represented for every single crop height). And this evolution can be identified on the georeferenced impact LiDAR return map generated by applying the proposed methodology (Figure 14). This figure allows clearly identify the single points in the crop row where leaf area decreases or increases substantially, according the representation of georeferenced map of density of LiDAR returns.

## 4. Discussion

Even in uniform vineyards, important differences in canopy dimensions (crop width and canopy volume) can be observed along the lines. The proposed method for processing and georeferencing the data obtained with a sensor LiDAR allows generation of digital canopy maps that can improve the crop management. Incorporation of GPS into the system results in greater accuracy of the obtained values. Even its use only for velocity recording has demonstrated its interest, in coincidence with [34]. GPS has also improved the procedure of assembling data from the two semi-canopy volumes obtained during the normal reading field process. This concept can avoid the need to assume the canopy structure based on a symmetric measurement of just one half of the canopy [25]. However, regardless of the sensor used, such canopy density maps must be defined according a reference grid to enable further comparisons.

The georeferencing data process allows exact placement of the generated map on the field. Such obtained results and the use of specific extended software for mapping and land image capture are in accordance with the conclusions obtained by Schumann *et al.* [25]. The data management process is neither easy nor quick. As a result, extra time for management and processing information is required prior to obtaining the results. Accordingly, methods of enabling real time generation of a canopy map are currently being investigated.

The combination of a LiDAR sensor and GPS receiver allows accurate information regarding canopy characteristics and its placement on the field to be obtained. However, further developments are needed to improve the accuracy of the results. Additionally, LiDAR height and LiDAR movements during field measurements can influence the precision of the created maps [29].

The proposed georeferencing method and density mapping is conducted in accordance with previous studies to characterize the canopy for further applications. Based on the spots cloud obtained with the LiDAR sensor, alternative methods proposed by different authors could be applied to determine the most important canopy parameters. By applying the procedure proposed by Walklate *et al*. [35], parameters such as the crop area index (CAI), tree area density (TAD) or tree area index (TAI) could be defined for each point in the map. The same procedure could be followed to determine the canopy area or canopy volume following the principle proposed by Schumann *et al.* [25], canopy volume or foliar surface according to Palacín *et al.* [21] or the total canopy volume by applying the procedure for assembling the two semi row measurements defined by Rosell *et al.* [24,29].

## 5. Further Implications

As mentioned in the introduction, improvement of pesticide applications can be achieved with detailed information regarding the crop structure. The use of such density maps appears to be an adequate tool for determining the most suitable volume rate for spray applications. New tendencies as variable application rate principle, on which canopy characteristics represent a key factor in the procedure to determine the optimal volume rate [1,4], could be improved by this proposed methodology. A good georeferenced canopy map could substitute with success the expensive and sophisticated method of canopy measurements using ultrasonic sensors. Another further implication of this application could affect the new alternative methods for dose expression in fruit and vineyard crops. Alternatives as Tree Row Volume (TRV) or Leaf Wall Area (LWA) concepts have been proposed recently in some international forums [36]. For those alternative methods, all the new developed methodologies for canopy characterization represent an important interest and increase the probability of success of this new dose expression concept.

Even more, the possibility to establish the georeferenced canopy map will derive in a complete development of the traceability concept. If pesticide dose is delivered according the canopy characteristics, the system will allow one to record on every single point on the parcel, the exact amount of PPP delivered, and consequently the potential risk of contamination on every zone on the parcel. One of the most immediate applications of this proposed methodology is to link the georeferenced maps with *Dosaviña*, the software developed by this research group [37]. This fact will derived on an automatic canopy data introduction into the developed system.

However, these maps are not only useful for pesticide applications. Indeed, the system generated herein could include real-time determination of vine canopy sizes as a tool to adapt different crop management processes (irrigation, fertilizer, pest control) or even to predict other information such as yield, labour needs, wood production, *etc*. Agreeing with the conclusions in [20], the use of those terrestrial laser scanning system offers a more rapid and systematic means of measuring tree crown structural properties, which are not easily obtained with traditional in situ methods, for agricultural monitoring and management. The development of such methodologies to describe canopy characteristics architecture proposes the idea that perhaps those systems should replace current laborious and time-consuming manual approaches, adapting the vine plantations for precision agriculture management.
